# Volatility, Uncertainty, Complexity, and Ambiguity (VUCA) in Healthcare

**DOI:** 10.3390/healthcare12070773

**Published:** 2024-04-02

**Authors:** Ana Cernega, Dragoș Nicolae Nicolescu, Marina Meleșcanu Imre, Alexandra Ripszky Totan, Andreea Letiția Arsene, Robert Sabiniu Șerban, Anca-Cristina Perpelea, Marina-Ionela (Ilie) Nedea, Silviu-Mirel Pițuru

**Affiliations:** 1 Department of Organization, Professional Legislation and Management of the Dental Office, Faculty of Dental Medicine, “Carol Davila” University of Medicine and Pharmacy, 17-23 Plevnei Street, 020021 Bucharest, Romania; robert.serban@umfcd.ro (R.S.Ș.); anca-cristina.perpelea@drd.umfcd.ro (A.-C.P.); silviu.pituru@umfcd.ro (S.-M.P.); 2 Department of Prosthodontics, Faculty of Dental Medicine, “Carol Davila” University of Medicine and Pharmacy, 17-23 Calea Plevnei, 010221 Bucharest, Romania; marina.imre@umfcd.ro; 3 Department of Biochemistry, Faculty of Dental Medicine, “Carol Davila” University of Medicine and Pharmacy, 17-23 Plevnei Street, 020021 Bucharest, Romania; alexandra.ripszky@umfcd.ro; 4 Departament of General and Pharmaceutical Microbiology, Faculty of Pharmacy, “Carol Davila” University of Medicine and Pharmacy, 6 Traian Vuia Street, 020956 Bucharest, Romania; andreea.arsene@umfcd.ro (A.L.A.); marina.nedea@umfcd.ro (M.-I.N.)

**Keywords:** VUCA, VUCA treatment, doctor-patient relation, healthcare system, management

## Abstract

Our professional activity is constantly under pressure from a multitude of elements and factors that can be classified into the four components of the VUCA phenomenon—volatility, uncertainty, complexity, and ambiguity—components that define the turbulence and challenges of the external environment. Considering the general elements of this phenomenon, we designed a new VUCA dimension specific to the healthcare field within which we have identified and analyzed all the factors that can influence the main actors of the doctor–patient relationship and the effects that can occur within the healthcare system in which this relationship is born. In this context, we generated the VUCA treatment in healthcare capable of mitigating the impact of this phenomenon; this treatment involves essential elements in overcoming possible crises and vulnerabilities of the medical profession. The VUCA treatment in healthcare requires combating volatility, uncertainty, complexity, and ambiguity through vision, understanding, clarity, and agility, which are grounded in the doctor’s need to acquire cross-functional competencies (soft skills). These competencies are applicable by using functional mechanisms and techniques that support the doctor in developing adaptability and anticipation skills, understanding the patient’s needs and addressing them, and ensuring the functionality and efficiency of the healthcare system by transferring these elements from micro-management to macro-management levels.

## 1. Introduction

The doctor–patient relationship represents the structural foundation of the existing healthcare system and is its most important constitutive cell. The importance of this relationship stems from the need to protect and preserve the main social and legislative value—human life and health [1]—but also because it represents the key point of origin for all other legal relationships specific to the healthcare field (doctor–patient, doctor–doctor, doctor–healthcare institution, patient–healthcare institution). Furthermore, the importance of the doctor–patient relationship is given by the legislative protection it benefits from, which methodologically structures its functionality from the perspective of rights and correlative obligations, as well as from the perspective of the normal management of this relationship and of the potential conflicts that may arise because of the intervention of professional error causing prejudice. Although the current legislation establishes a comprehensive framework regarding patient rights and correlative obligations to be respected by the doctor, we must be aware of the entire spectrum of factors that can influence, to varying measures, the quality of medical services provided for the benefit of the patient.

### The VUCA Concept—Volatility, Uncertainty, Complexity, and Ambiguity of the Surrounding World

The world in which we live and conduct our professional activities represents the current context to which we must continuously adapt. It is characterized by the influence of a multitude of factors that can be categorized into volatile, uncertain, complex, and ambiguous elements. These four terms form the basis of the acronym VUCA, which encapsulates the entirety of challenges in the external environment [2], which is an environment that defies certainty and confuses the population [3].

The VUCA acronym was introduced by the US Army to describe the turbulence of global systems following the end of the Cold War [2,4,5,6]. VUCA changes the rules during the game, making it difficult to react immediately due to frequent and dynamic changes. This concept has also been adopted in business, organizational management, and leadership, expanding into various areas. At its core, VUCA is a challenging view of reality. It incorporates all the influences and factors that can intervene and affect both the private and professional environment. The VUCA concept has found its place in almost everything around us, from government policy to people’s daily choices and decisions. It has been used to guide institutions and individuals to be less affected when faced with complex, unknown, and unseen phenomena [4]. To understand the influences of the concept, we present below a description and detailed breakdown of the specific characteristics of each element of this acronym.

The specialized literature describes *volatility* as a characteristic of an unstable and unpredictable situation [3], and the ability of something to change rapidly and unpredictably [5,6,7], which signifies availability and accessibility to information and data, as well as the appearance of understanding the current situation, yet changes occur too frequently and are hard to forecast [3]. 

The *uncertainty* of the world we live in refers to situations or events that are not clear, and for which we do not have sufficient information given the short time elapsed since their occurrence [6]. Uncertainty is also characterized by an overwhelming sense of not knowing what changes might occur in the near future [3,8]. The Organisation for Economic Co-operation and Development (OECD) describes uncertainty from the perspective of the quality of information we have regarding the occurrence of a certain event/situation, as well as the inability to anticipate a possible outcome prior to the triggering of this event [5]. 

*Complexity* must be understood from the perspective of a multitude of interconnected parts that form an elaborate network of information and processes; these are often multiform and intricate, which, due to the intellectual, emotional, and physical burden, render individuals incapable of implementing all these processes within the imposed deadlines and conditions [3]. Thus, complexity arises from the need to react appropriately to find ways to adjust and coordinate actions and strategy according to the elaborate system of information available [9].

*Ambiguity*—If uncertainty refers to the lack of information that makes one unable to develop a zone of predictability regarding the future, ambiguity implies the existence of an unclear reality due to mixed meanings of conditions [8], meanings that can generate multiple interpretations. Ambiguity arises when an event, situation, or context is unclear [6], either because information is lacking, inconsistent, contradictory, or somehow obscure [5], or because it is interpreted differently by specialists in the same field, with these interpretations subsequently conveyed to the general public.

In terms of examples of VUCA events, Professor Miguel Ángel Ariño of IESE Business School, University of Navarra, states that humanity has experienced at least three events that have triggered the full spectrum of VUCA and that made the global population feel vulnerable and out of control: the terrorist attack on 11 September 2001, the financial crisis of 2008, and the COVID-19 pandemic [10]. VUCA has manifested in the following ways:

- *Volatility*—the production costs of basic goods have continuously fluctuated, leading to financial fluctuations in terms of tariffs for goods and services provided;
- *Uncertainty*—there have been insights into understanding how events were triggered, but there is no certainty as to when and in what context they might recur;
- *Complexity*—there has been an uncontrollable accumulation of variables that have complicated the normal and known way of doing things (costs, tariffs, legislation, people, etc.);
- *Ambiguity*—there were too many sources of information about events and their consequences, and that presented different perspectives of reality and multiple interpretations, which induced ambiguity in the global population [3,10].

## 2. Materials and Methods

This article is based on research conducted between April 2023 and January 2024. We utilized the VUCA concept (volatility, uncertainty, complexity, and ambiguity) as the foundational framework and analytical tool of the research. While VUCA has been studied in various areas and fields of activity [11], it is the first time that a specific dimension of VUCA is constructed for the healthcare domain, with the aim of identifying, analyzing and acknowledging the multitude of potentially conflicting elements. These are both internal and external factors capable of influencing the proper and effective management of the doctor–patient relationship and its broader impact on the healthcare system. We analyzed each element of this acronym to outline a comprehensive picture of the concept, but also to understand how it can be addressed. It is important to note that the study conducted does not have a null hypothesis, so the specific elements of the VUCA concept—volatility, uncertainty, complexity, and ambiguity—are normal and specific characteristics of the healthcare ecosystem.

Thus, another innovative element of this article is the generation of a functional treatment—the VUCA treatment in healthcare—which can be implemented to mitigate the negative dimension of VUCA. This treatment serves as a guide to promote healthcare stakeholders towards greater awareness, preparedness, resilience, and the development of an anticipatory approach to facing the uncertainties of future challenges.

**Figure 1:**
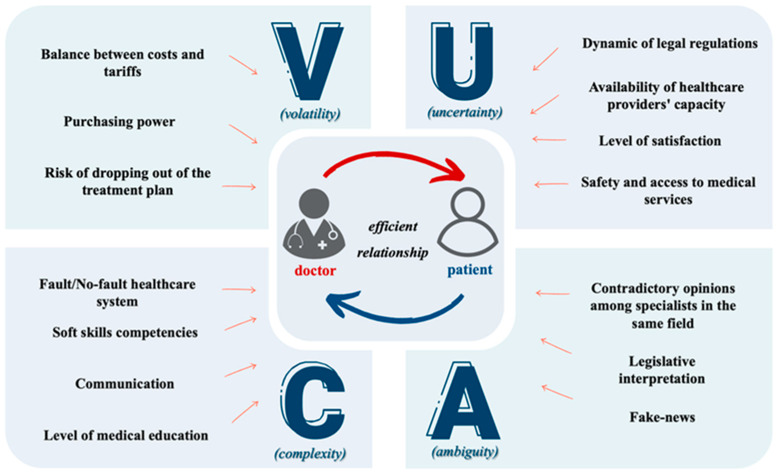
Doctor–patient relationship in VUCA context.

Building on the general elements discussed above, we present below a new innovative approach and dimension of the VUCA concept—**VUCA in healthcare**—defining it from the perspective of the doctor–patient relationship, which is a relationship that develops within a complex healthcare system (Figure 1). We identified and analyzed all factors that fall under the elements of this acronym to build the real context of medical practice and to raise awareness of the continuous need for learning and adaptation.

## 3. Results

### 3.1. VUCA in Healthcare

#### 3.1.1. Volatile Elements in Healthcare

Volatility refers to a strong financial and economic component, which thus intervenes from the perspective of **costs and tariffs applied in healthcare**. This volatile element must be analyzed both from the perspective of the doctor and from the perspective of the patient as the main beneficiary of healthcare services.

By costs, we refer to the totality of individualized expenses attributed to a product/service, which can be categorized into direct and indirect costs. The following can be identified: expenses for materials and medications, human resources (medical and administrative staff), maintenance [12], rent, equipment and its servicing, etc. 

The tariff can be understood as the actual price paid by the patient, which is usually set as the average cost per patient/medical act/period of medical care [13]. This financial element must consider the efficiency of providers regarding the desired medical outcome, the quality and access of patients to medical services [14], the relative amount of time of the physician, the level of training and experience in providing a particular service, and the costs associated with operating a medical office/facility [5]. The tariff often includes “other related benefits” provided to the patient, such as the location of the medical office (city center), proximity to public transportation, availability of parking spaces and the comfort provided by the layout and setup of the space. 

The continuously volatile nature of the existing ecosystem has the capacity to jeopardize the doctor–patient relationship through the lens of financial fluctuations brought about by the numerous crises humanity has experienced. Thus, the continuous increase in monetary values specific to the elements constituting the costs associated with managing a medical practice/healthcare facility (rent, utilities, materials, etc.), will shape another, larger dimension of the tariffs applied to ensure the provision of medical services. This aspect puts pressure on the physician in terms of his ability to survive these financial fluctuations.

At the same time, we can also consider the impact it can have on the patient, who is also affected by these volatile effects, in terms of the **“purchasing power”** for medical services because of the rising tariffs. This aspect is corroborated by a survey conducted by the U.S. Physicians Foundation in 2016, which indicates that patients are increasingly concerned that they will not be able to afford necessary care, as medical costs continue to rise [15]. Therefore, there is an imminent risk that the previously established and agreed treatment plan will be abandoned [16], leading to a gradual and accentuated deterioration of the patient’s state of health, and subsequently to higher additional costs to restore optimal health conditions.

#### 3.1.2. Uncertain Elements in Healthcare

One element characterized by a strong influence of uncertainty is **the dynamic cycle of legal regulation**, especially when analyzed from the perspective of the health sector. In many situations characterized by a major or emerging health threat, policymakers with legislative competence adopt regulatory acts in the absence of complete evidence or scientific certainty, driven by the need for an urgent response and perhaps the need to construct an innovative approach [17]. 

An example of this is the COVID-19 pandemic, which has led to a continuous cascade of legislative acts in too short a period of time to ensure effective communication, transparency, and the correct application of legal norms. This aspect had the potential to jeopardize healthcare professionals, patients, and the community as a whole, given the lack of clarity and uniformity of approach to possible future events and consequences. As a result, the dynamic nature of legislation brings with it a dose of complexity for healthcare professionals and for the healthcare system, in terms of the constant and rapid need to adapt to the new and to comply with regulatory requirements in order to avoid the possibility of subsequent sanctions. From a strategic point of view, information and its correct transmission, in terms of competence and quality, are the essential sources for reducing uncertainty, extending to a complex approach based on the analysis of multiple perspectives [3]. 

Returning to the volatile element described in the previous sub-section, one effect of economic volatility could be represented by the reduced ability to estimate and anticipate **the availability of a healthcare provider’s capacity**, and the associated uncertainty. This uncertainty can have a significant impact on the functioning of the doctor–patient relationship and the provision of healthcare services, including in terms of complicating the management process of the medical practice/healthcare facility. Increased costs are an element that can affect the work of healthcare providers, which, as mentioned above, can be caused by suppliers of medical materials, equipment, and products. Uncertainty and the inability to anticipate possible events that could affect stocks, in terms of not being able to provide them in the long term due to financial powerlessness or sudden high demand on the market, will affect the work of medical staff, who will not be able to provide health services as needed or requested. Therefore, from the patient’s point of view, we can discuss the uncertainty induced in them regarding the **safety and access to medical services**. Access to medical services refers to the ease with which individuals can obtain necessary healthcare [18], also defined as the opportunity to use appropriate services proportional to healthcare needs [19,20]. 

In particular, uncertainty about the safety and accessibility of medical services was widely felt during the COVID-19 pandemic, which brought with it several barriers that “inhibited” access to medical care (financial, spatial, temporal, informational, human, and technological) [18]. 

#### 3.1.3. Complex Elements in Healthcare

If we consider the *healthcare system* as an ecosystem of all the relationships that develop around the notion of “health”, we cannot overlook the complexity that the nature of the system brings, depending on the legal approach it adopts in managing allegations of malpractice. It is important to mention that this legislative approach helps to shape the mindset of the community in which it is implemented. Thus, there are two types of healthcare systems—*the fault system* and *the no-fault system*.

The two systems can be distinguished by the following characteristics:

- *The no-fault system*—The mechanism used in this system prioritizes the rapid compensation of patients harmed by medical acts, without the need to prove the guilt of the culprit, and promotes the idea of identifying the error and the conditions in which it occurred, in order to establish rules that would anticipate future error [21]. This system focuses on implementing a compensation model at a national level [22]. Countries such as Sweden, Norway, Denmark, and New Zealand have implemented such a system, which emphasizes extrajudicial procedures. This system has proven to be useful, as evidenced by the small number of malpractice cases that are resolved in court [23].
- *The fault system*—This system is used in the USA, most European Union states, and Romania. The compensation process for patients can be cumbersome due to the obligation to pursue and prove specific malpractice conditions, including the illicit act, the damage, the causal link, and the guilt [21,24]. Although mediation procedures are regulated by law, the community’s focus is primarily on punishing the presumed guilty party rather than compensating the harmed party. It is important to note that this mindset may hinder the effectiveness of mediation as a means of resolving disputes.

In the no-fault system, doctors feel safe reporting errors [25]. However, in the fault-based system, doctors’ activities become complicated, which has significant repercussions on their professional safety. The impact of this system complexity on the doctor–patient relationship must be analyzed objectively, considering the patient’s role in initiating legal proceedings. According to the law, only the harmed party or their representative has the legal capacity to file a lawsuit, request the physician’s sanction, and seek compensation for the damages incurred. Malpractice accusations are frequently dismissed by the court due to the absence of legal conditions for initiating the procedure. However, if the process is initiated, it can have a significant impact on various aspects of the doctor’s life, directly affecting their professional future.

This may occur due to *patients’ different perceptions of healthcare and their need for medical education* based on their own values and knowledge, which may lead to a lack of awareness of the complexity of medical procedures, the time and effort required, and the risks involved in resolving a medical case. As noted above, a potential solution to this problem would be for doctors to allocate more time to educating patients, thus becoming the primary providers of information [26,27]. As a result, the complexity of medical practice is constantly increasing. Therefore, physicians must continuously strive to navigate the learning curve, not only in terms of medical expertise, but also in acquiring *transversal skills and competencies* (soft skills), to effectively manage all legislative, economic, situational, and relational challenges. Transversal skills refer to a set of personal qualities, habits, attitudes, and social behaviors that make someone a good employee/collaborator and a compatible person to work with [28]. 

Research conducted by Harvard University, along with the Carnegie Foundation and the Stanford Research Center, has concluded that 85% of workplace success is attributed to soft skills, while only 15% of success is the result of professional skills (hard skills) [29]. In the medical field, *communication* is a highly important soft skill that ensures efficient horizontal relationships (doctor–patient, doctor–doctor) and vertical relationships (doctor–medical facility, doctor–government institution) [30]. It is true that mastering the elements specific to effective communication requires additional effort on the part of medical personnel. However, applying this knowledge would have a positive effect on treatment outcomes by correctly identifying areas of vulnerability and effective ways to improve health [31]. 

#### 3.1.4. Ambiguous Elements in Healthcare

In healthcare, the *influence of mass media* can introduce ambiguity into the doctor–patient relationship [32]. From a certain perspective, we are referring to the abundance of information available on various media portals and patients’ intentions to access and interpret what they find based on their own values, knowledge, and competencies. A study conducted in the USA confirmed this, finding that 80% of Americans search the internet for health-related topics [33]. From another perspective, we consider the impact that different media sources can have on the transmission of information. In their quest for catchy slogans, sometimes without sound statistical data [34], they construct a distorted dimension of reality for both doctors and patients.

The issue lies in the formation of different and inaccurate perceptions of health by the patient and the doctor’s efforts to confront these perceptions in order to improve the patient’s state of health. We therefore reiterate the importance and the dual role of the doctor. In this context, in addition to the mission of treating the patient, the doctor must establish an effective relationship with the patient in order to develop a sense of trust in the competence and skills of the medical staff. This relationship is based on the doctor’s second mission, which is to continuously educate the patient [26,27], since the doctor is the only correct and certified source of medical information.

Another dimension of ambiguity can be seen through the lens of *contradictory opinions generated by healthcare specialists* working in the same field on a given medical topic. This generates the patient’s need for safety because of the uncertainty, lack of confidence, and doubt that has been induced in them.

Ambiguity is a characteristic that can also be generated by the uncertainties of existing legal acts, which stimulate *different legal interpretations by lawyers*, due to the great interest they have in the field of healthcare, especially regarding potential malpractice situations. Their interest in this area derives from the legal nature and importance of the protected value—human life and health. Lawyers could be seen as indirect promoters of the phenomenon of defensive practice, through the hostile attitude they display in advocating the immediate blaming and sanctioning of doctors in front of the court and the whole community. This attitude creates a profound state of fear among medical staff who, as a result of the increasing number of malpractice allegations (justified and unjustified), will react defensively by refusing to provide medical services or recommending unnecessary additional tests for fear of future malpractice allegations [35]. Consequently, this leads to an increased state of vulnerability among patients by limiting their access to health services.

## 4. Discussion

**Figure 2:**
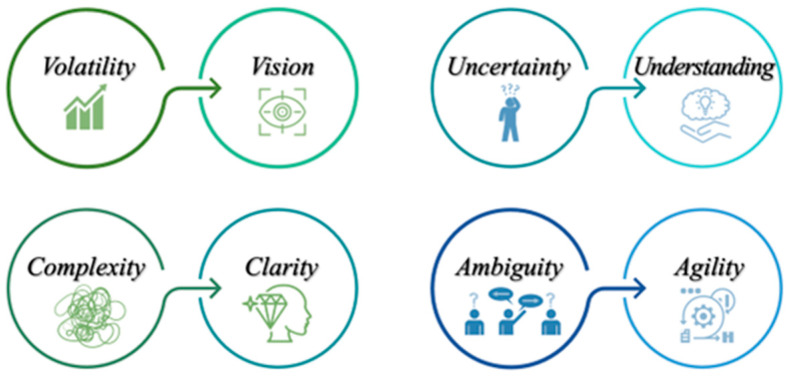
Elements to combat the negative dimension of VUCA.

However, it is important to note that VUCA should not be viewed solely from a negative perspective. As previously observed, VUCA has been identified, experienced, and recognized throughout various historical periods and from different perspectives. Ultimately, it highlights the need to formulate a positive approach to the four elements, transforming volatility into vision, uncertainty into understanding, complexity into clarity, and ambiguity into agility (Figure 2) [2,4,6,36]. 

From a functional perspective, addressing volatility requires a clear, tangible, and strategic *vision* as a criterion for making informed decisions [6,36]. This involves coordinating efforts, investments, and material and human resources across all areas and levels [4].

Clear *understanding* of the existing situation can overcome uncertainty [6]. Understanding refers to how leaders and managers perceive and listen to aspects related to the expertise and functional components of their business and activities. This helps to identify vulnerabilities, comprehend them, and develop a vision to overcome them [4]. It also involves being aware of different perspectives within various scenarios [36]. 

To address complexity, it is recommended to *clarify* events that are difficult to understand and to adopt a strategy based on simplicity. This approach can enhance capacity, flexibility, and tolerance, which can help to mitigate complexity [4].

*Agility* represents the response to ambiguity. It is the ability to respond quickly to unforeseen changes [2], enabling prompt decision making and action [6]. Agility is closely linked to learning and speed, and involves the rapid development of solutions through continuous learning, rejection, and redefinition of proposed solutions [4]. 

### 4.1. VUCA Treatment in Healthcare

The VUCA treatment in healthcare has a distinct level of specificity. Positive elements such as vision, understanding, clarity, and agility should be considered in terms of practical mechanisms that can be applied and utilized in the doctor–patient relationship. The mechanisms should address how doctors can use VUCA to their advantage. They must be easily accessible to the doctor, who is the primary and active participant in the healthcare system, with legal and educational responsibilities for effectively managing the relationship with patients.

The management sphere provides several functional solutions (micro-/macro-/self-management) that require the acquisition of transversal competences in addition to professional skills. These transversal competences enhance professionals’ adaptability and differentiate them from other specialists in the same field in terms of competitiveness. They provide a perspective for efficiently managing material, financial, human resources, and the most valuable resource—time.

#### 4.1.1. Vision in Healthcare

Vision is the result of the *development of analytical thinking* in both individuals and professionals, which implies a profound need for clarity in assessing the current context, and individual and community social deficits and needs, with the aim of applying specific techniques and methods of creativity to innovate and address these needs.

Technically, doctors can apply *SWOT analysis* to their own professional activities to create a clear vision based on a strategy that correlates all available or potential resources. SWOT analysis is a method for identifying and assessing strengths, weaknesses, opportunities, and threats [37]. SWOT analysis enhances the awareness of key elements and their utilization in building a future behavioral approach. In the first part of this article, factors that may affect the doctor–patient relationship were identified and analyzed. These factors were classified as constitutive elements of VUCA and represent weaknesses and threats in the SWOT analysis. Identifying these elements enables the physician to determine which are the strongest or weakest points of the medical activity.

**Figure 3:**
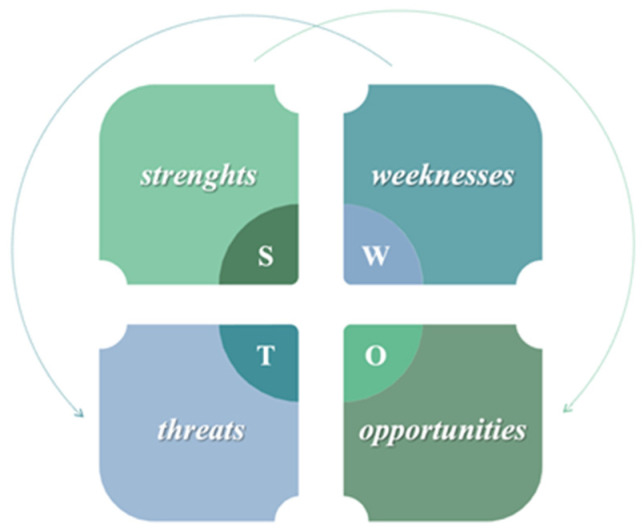
Graphic representation of SWOT analysis.

From a temporal perspective, strengths and weaknesses are static concepts of the present based on descriptive parameters of activities carried out within a specific period of time. It is important to be aware of these aspects in terms of their potential future impact. Thus, during times of crisis, weaknesses can become threats that need to be addressed, while strengths represent opportunities that enable and drive the mitigation of the crisis that has been triggered (Figure 3).

#### 4.1.2. Understanding in Healthcare

Understanding supports vision by correctly grasping the characteristics of the present. Understanding can be achieved through the assimilation of *problem-solving* competence, which highlights an individual’s ability to identify the root cause of a problem, generate viable solutions, monitor and improve processes, and thereby prevent the occurrence and repetition of similar problematic situations in the future. Therefore, it is not enough to simply define the multitude of existing events, but also to be aware of the root cause of their occurrence.

One of the techniques that can be used for this purpose is the “5 Whys” technique—a management method used to identify the root cause of problems that could be used to build the “understanding” dimension in VUCA. This technique involves repeatedly asking “why?” until the real cause of the problem is identified [38]. The technique focuses on identifying the incorrect process or mechanism that triggered the problem, rather than blaming an individual.

In the context of the doctor–patient relationship, when a conflict arises, the doctor can use this technique to identify the triggering cause and improve the conflict situation with the patient. The technique also facilitates the prevention and anticipation of possible conflicts by adapting one’s behavior and activities to new processes and procedures, which are elements of the future strategic vision.

#### 4.1.3. Clarity in Healthcare

As seen earlier, clarity alleviates complexity by simplifying processes. Clarity in the doctor–patient relationship can be developed by ensuring *effective communication* based on the *emotional intelligence* of medical staff.

Returning to the multitude of VUCA factors that can disrupt the relationship, when understanding provides the context and vision develops the strategy for the future, the clarity provided by effective doctor–patient communication helps to mediate and resolve the vulnerabilities that arise.

In schematic terms, the physician has the competencies and skills necessary to identify the vulnerabilities created by VUCA, understand their specificity, and identify a viable treatment. The treatment is applied by providing the patient with adapted information through accurate information, which contributes to increasing the patient’s level of education in terms of adopting a positive attitude towards prevention, increasing the patient’s level of acceptance and fostering trust in their doctor.

**Figure 4:**
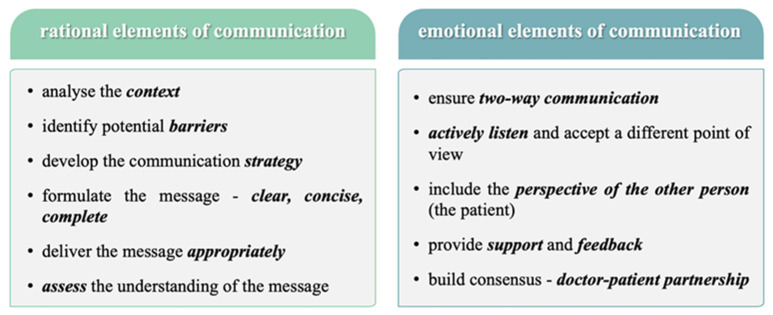
Rational and emotional elements of communication process.

The physician must recognize that he is the only actor responsible for the effectiveness of communication and must apply the essential elements of an effective communication process, which are both rational and emotional (Figure 4).

This ability to communicate effectively is supported by the development of *leadership and social influence skills* that can be cultivated, assimilated, and learned. This includes the ability to manage, develop, and lead people and teams by creating visions, shaping values, defining strategies, and ensuring unity for effective transitions during change.

From the perspective of the doctor–patient relationship and its purpose, the process of improving the patient’s health is likely to generate change and resistance to the doctor’s recommendations, as the patient may find it difficult to accept their own health status and the proposed treatment. In this context, leadership and social influence support the second component of the doctor’s professional activity—patient education—which can be achieved through guidance and counseling, with the doctor acting as the conductor of this relationship based on his professional competencies.

Furthermore, in the context of accentuated digitalization through technological progress, represented by the development of disruptive innovations, there is a need for professionals to acquire specific competences in the use, *monitoring, and control of technology*, as well as in its *design and programming*. This is a prerequisite for a high level of professional security for the physician, as well as responding to the emotional and rational needs of the patient, thus generating trust in the professional and greater acceptance of the proposed treatment. Sir William Osler (1849–1919), a Canadian physician and one of the “Big Four” founding professors of Johns Hopkins Hospital, once said: *“The good physician treats the disease; the great physician treats the patient who has the disease.”* [39]. This statement, made over a century ago, emphasizes the importance and multidimensionality of the medical profession. It reaffirms its role in the relationship with the patient and within the healthcare system.

#### 4.1.4. Agility in Healthcare

Agility is the antidote to VUCA ambiguity, covering the lack of clarity regarding events. As we have seen, agility refers to the need for immediate response based on the continuous process of *learning and acquiring competencies and skills*.

In the field of learning and knowledge acquisition, the term “obsolescence”, borrowed from nuclear physics, has been used for some time to describe the concept of halving time, a decreasing process that involves the loss of acquired knowledge and the failure to acquire new knowledge. This happens when individuals do not make constant efforts to repair informational erosion, innovate, and grow intellectually [40]. Obsolescence applies to all fields of activity, including healthcare. Technological advances and ongoing discoveries contribute to the expansion of the information landscape and the replacement of elements and information that are no longer current. This increases the need for individuals to continually update their knowledge and skills in order to maintain their position in the field of activity.

In 1971, Professor J. Lukasiewicz of Carleton University in Ottawa stated that the information acquired by an engineering graduate in 1940 remained useful and applicable for twelve years. However, this period had been reduced to five years over a period of 30 years. In the same year (1971), Dr Edward C. Rosenaw Jr, vice-president of the American College of Physicians, estimated that medical knowledge would remain useful and applicable for up to five years [40]. In 2017, Martín-J. Sepúlveda, visiting professor at Harvard University, said the novelty and usefulness of medical knowledge decreases, citing an applicability period of 18–24 months [41]. 

The constant compression of these periods of knowledge application leads to the need to implement the concept of *“learn, unlearn, relearn”* [42]. Writer Alvin Toffler said: *“The illiterate of the 21st century will not be those who cannot read and write, but those who cannot learn, unlearn and relearn.”* [43]. This cycle is essential in a VUCA world characterized by multiple and rapid changes and must be applied continuously to ensure the continuity and relevance of the knowledge and skills acquired and applied in professional activity.

From the perspective of the professional and the actor responsible for the success of the relationship with his patient, the doctor must focus on *active learning and the application of specific and tailored learning strategies*. This involves minimizing the risk of triggering professional and intellectual self-sufficiency by continuously traversing the learning curve from the stage of unconscious incompetence to unconscious competence. It is important to be aware of the dynamics of the world around us, and how it is being reconfigured and restructured, and to apply adapted techniques that make learning an enjoyable, efficient, and continuous process. 

## 5. Conclusions

**Figure 5:**
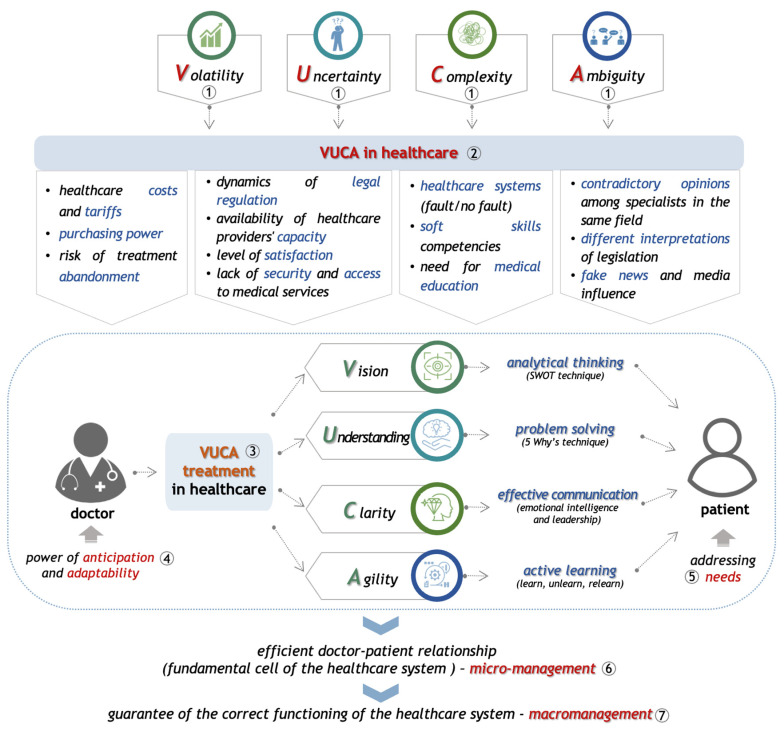
Step-by-step process of the VUCA structure in healthcare: ① general VUCA characteristics; ② identification of VUCA dimension in healthcare; ③ implementation of VUCA treatment in healthcare; ④ the impact on medical staff; ⑤ the impact on patients; ⑥ the impact on doctor-patient relationship; ⑦ the impact on the healthcare system.

Schematically, the construction of the VUCA phenomenon in healthcare and its treatment can be achieved as follows (Figure 5):

1. Understanding the nature and functionality of the general characteristics of the constituent elements of VUCA—volatility, uncertainty, complexity, and ambiguity.
2. Healthcare professionals must have the necessary knowledge, skills, and abilities to be aware of the specificity of the VUCA phenomenon in healthcare. The ***new VUCA dimension in healthcare*** thus represents an accumulation of elements and factors capable of influencing the professional activity of medical staff, the quality of the medical services provided, and the effectiveness of the relationship they build with their patients, with significant implications for the healthcare system as a whole.
3. In order to mitigate the effects of this phenomenon, the doctor will apply the ***VUCA treatment in healthcare***, which is a functional guide based on principles from which arises the need to acquire cross-functional competencies, whose applicability can be achieved through the use of efficient mechanisms and techniques capable of clarifying and reducing the crises and chaos created by VUCA. This can be achieved as follows:
  ▪ Volatility will be countered by vision, which the doctor can achieve by developing analytical thinking; this defines his ability to understand the real context in which he operates, using the SWOT technique;
  ▪ Uncertainty will be replaced by understanding, by developing the ability to solve problems efficiently, using techniques to identify the root cause and reduce the likelihood of recurrence, such as the “5 Why’s” technique;
  ▪ Complexity is replaced by clarity, based on the doctor’s ability to communicate effectively (to listen and be understood) as an essential characteristic in the development of emotional intelligence and specific leadership skills to be applied in the relationship with the patient;
  ▪ Ambiguity is replaced by the doctor’s agility, which is the result of the effort of active learning, ensuring a continuous process of “learning, unlearning, relearning”.
4. Therefore, if we consider that VUCA is a certainty of the contemporary world that is intensifying year by year, the application of this treatment proves to be a necessity in the performance of the profession. This treatment ultimately supports the professional in developing two essential characteristics: anticipation and adaptability.
5. This, in turn, offers the perspective of better understanding and addressing more easily the needs of the patient, as the real beneficiary of the health services and of all the efforts made at the system level.
6. In this context, we can consider the doctor–patient relationship as efficient, representing the fundamental cell of the healthcare system, from the perspective of successful micro-management.
7. This is therefore a guarantee of the correct functioning of the entire system at the level of macro-management applied to healthcare.

## Data Availability

No new data were created or analyzed in this study. Data sharing is not applicable to this article.
